# Experiences after Twenty Months with Pandemic Influenza A (H1N1) 2009 Infection in the Naïve Norwegian Pig Population

**DOI:** 10.1155/2011/206975

**Published:** 2012-01-03

**Authors:** B. Gjerset, C. Er, S. Løtvedt, A. Jørgensen, O. Hungnes, B. Lium, A. Germundsson

**Affiliations:** ^1^Department of Laboratory Services, Norwegian Veterinary Institute, P.O. Box 750 Sentrum, 0106 Oslo, Norway; ^2^Department of Health Surveillance, Norwegian Veterinary Institute, P.O. Box 750 Sentrum, 0106 Oslo, Norway; ^3^Department of Control, Norwegian Food Safety Authority, Kyrkjevegen 332, 4325 Sandnes, Norway; ^4^Norwegian Pig Health Service, Animalia, P.O. Box 396 Økern, 0513 Oslo, Norway; ^5^Division of Infectious Disease Control, Department of Virology, Norwegian Institute of Public Health, P.O. Box 4404 Nydalen, 0403 Oslo, Norway

## Abstract

Pandemic (H1N1) 2009 influenza A virus was detected in Norwegian pigs in October 2009. Until then, Norway was regarded free of swine influenza. Intensified screening revealed 91 positive herds within three months. The virus was rapidly transmitted to the susceptible population, including closed breeding herds with high biosecurity. Humans were important for the introduction as well as spread of the virus to pigs. Mild or no clinical signs were observed in infected pigs. Surveillance of SIV in 2010 revealed that 41% of all the Norwegian pig herds had antibodies to pandemic (H1N1) 2009 virus. Furthermore, this surveillance indicated that pigs born in positive herds after the active phase did not seroconvert, suggesting no ongoing infection in the herds. However, results from surveillance in 2011 show a continuing spread of the infection in many herds, either caused by new introduction or by virus circulation since 2009.

## 1. Introduction

Swine influenza A viruses (SIVs) are enzootic in most European pig populations [1]. The SIV H1N1 and H3N2 subtypes have been circulating for more than 30 years, and the SIV subtype H1N2 has been circulating since it was first isolated in Great Britain in 1994 [2, 3]. In April 2009, a new influenza A virus of subtype H1N1 emerged in the human population in Mexico and the United States. This was a multiple reassortant virus containing genes from North American and Eurasian lineages [4, 5]. The virus spread rapidly across the world by human-to-human transmission. Human-to-pig transmission was first reported in Canada in late April 2009, then in European pig population in early September 2009, and has since been reported in several countries all over the world [6–9]. In October 2009, SIV was reported for the first time in Norway when an integrated pig herd tested positive for pandemic (H1N1) 2009 virus after showing mild clinical signs of respiratory disease [10].

 The clinical picture of pandemic (H1N1) 2009 infections in experimentally and naturally infected pigs was described as mild respiratory illness, increased temperature, and decreased appetite [6, 11, 12]. In some infected herds, clinical signs were not detected [13]. Studies have shown that immunological naïve pigs experimentally infected with pandemic (H1N1) 2009 virus could transmit the virus efficiently to other naïve pigs [11, 14, 15]. Although pandemic (H1N1) 2009 virus contains gene segment genetically related to other swine influenza virus strains circulating in Europe and North America, it shows antigenetic differences in the major glycoproteins of the virus [4]. However, it has been shown that pigs infected or vaccinated with H1N1 European avian-like swine influenza virus could produce cross-reactive antibodies against pandemic (H1N1) 2009 virus which protected pigs against the infection of pandemic (H1N1) 2009 virus [13, 16].

 The pig production in Norway consists of approximately 2500 herds, mainly small family farms, with about 1.5 million slaughtered pigs in 2010. National surveillance and control program shows that the pig population is free for Aujeszky's disease (AD), transmissible gastroenteritis (TGE), porcine respiratory corona virus (PRCV), porcine reproductive and respiratory syndrome (PRRS), and *Mycoplasma hyopneumoniae* [17, 18]. The Norwegian Food Safety Authority has been running annual surveillance programs to document the status since 1994. SIV was added to the program in 1997. Under the surveillance, blood samples from about 500 randomly selected herds are collected annually for specific disease surveillance [17]. The surveillance has never detected SIV infection in Norway until October 2009, except for pigs in one herd that tested seropositive for subtype H3N2 in 1998 without showing clinical signs or further spread of the infection.

 This paper consists of studies that describe the initial spread of pandemic (H1N1) 2009 virus amongst Norwegian pig herds, the control measures initiated, and the infection status 20 months after the introduction. The paper also discusses the manifestation of the infection in the Norwegian pigs given that they had no prior immunity to any influenza A viruses and are free from most other viral respiratory diseases.

## 2. Materials and Methods

### 2.1. Study Material

Following the primary outbreak in October 2009, an intensified surveillance was immediately initiated to investigate the extent of SIV infection in the pig population in Norway, referred to as study 1 in the results section. The study targeted the following categories of herds: (i) herds where pigs with influenza-like signs were observed, (ii) herds where the pigs had been exposed to humans with verified pandemic (H1N1) 2009 virus infection or influenza-like illness (ILI), and (iii) herds with a history of contact with or in close proximity to infected herds through transfer of animals, usage of common equipment, for example, transport vehicles to slaughterhouse, and visitations from farmers of infected farms. In herds suspected to have an active infection, nasal swabs were collected from 20 pigs at different ages, with priority of sampling pigs with clinical symptoms. In herds without clinical signs, blood samples were collected from at least 20 pigs (sows or slaughtered pigs) for serological testing. Calculation of sample size was done assuming 100% test sensitivity and a within-herd prevalence >15%. The sample size implies that the risk of having 20 nonreactors from a positive farm is less than 4%.

 During the follow-up study, there was a special focus on the prevalence in nucleus and multiplier herds, hereafter referred to as study 2. The nucleus herds are closed herds meaning that they have no contact with other herds except via semen from the Norsvin AI station. The multiplying herds are either closed or obtain replacement sows from a single nucleus herd. They can sell sows to one or several conventional herds. The biosecurity level is high in both types of herds. Serological surveillance was carried out to investigate the possible spread of pandemic (H1N1) 2009 to these herds in the early phase of the outbreak. Between 1st of October 2009 and 31st of January 2010, blood samples were collected from 20 slaughtered pigs from 112 nucleus and multiplier herds.

 A third study was initiated to follow the course of the pandemic (H1N1) 2009 infection. From the herds that tested positive for pandemic (H1N1) 2009 in 2009, 38 herds consisting of 13 nucleus herds, 20 multiplier herds, and five conventional integrated herds were selected for the study. From each herd, blood samples from 20 slaughtered pigs were tested for antibodies against pandemic (H1N1) 2009. Blood samples were taken on the condition that 6 to 12 months had elapsed since the herd first tested positive and that the pigs tested were born at least 2 months after that time. An additional testing was performed in 9 herds about 18 months after the pandemic (H1N1) 2009 introduction.

 Study 4 consists of data from the national surveillance programme for SI in Norwegian pigs in 2009 and 2010. The program includes all nucleus and multiplier breeding herds and the nucleus unit of all sow pools. In addition, a random selection of conventional pig herds, 300 integrated and piglet-producing herds and 60 fattening herds, was selected from a population of approximately 2500 herds. Ten pigs in each herd were tested. With the exception of pigs from finishing herds, which are sampled at the abattoirs, the blood samples are collected from sows in the herds.

### 2.2. Clinical and Epidemiological Information

Clinical and epidemiological information recorded by The Food Safety Authority was available from 43 pig herds suspected to be infected. The information was collected, while the herd was being sampled during the early phase of the outbreak between 10th of October 2009 and 31st of December 2009. The selection of herds was based on the same criteria as described previously. The size of these herds ranged in numbers from 5 to 2000 pigs (average 550 pigs). The investigation used a structured questionnaire consisting of questions on epidemiological information including clinical picture within the herd, likely source of infection, contact with people diagnosed with pandemic influenza or had ILI symptoms and being in contact with pigs while sick, movement of animals in or out of the farm, and handling and biosecurity measures.

### 2.3. Serological and Virological Investigations

Initially, serum samples were analyzed for specific antibodies against A/Sw/Belgium/1/98(H1N1) and A/Sw/Flanders/1/98(H3N2) using hemagglutination-inhibition (HI) assays according to the method described in the OIE Manual of Diagnostic Tests and Vaccines for Terrestrial Animals [19]. Following introduction of pandemic (H1N1) 2009 virus in the Norwegian pig population, serum samples were tested for influenza-A-specific NP antibodies using an ELISA kit (ID Screen Influenza A Antibody Competition test, ID VET, Montpellier, France). A herd was considered seropositive if three or more blood samples from the herd were positive for influenza-A-specific NP antibodies. In case one or two of the 20 blood samples from a herd were ELISA positive, the herd was retested with samples from another 20 pigs, and the herd concluded positive if at least one of these samples was seropositive. Positive and/or inconclusive samples were retested for hemagglutinin-specific antibodies using HI assay with A/Sw/Belgium/1/98(H1N1), A/Sw/Flanders/1/98(H3N2), and A/California/07/2009 (pandemic (H1N1) 2009) as antigens. The antigens for the HI tests were produced in chicken eggs at the Norwegian Veterinary Institute in Oslo. Titers ≥40 were considered positive.

 Nasal swabs (Copan Innovation LTD, Brescia, Italy), individual or in pools of two, were placed in 1 mL of transport medium (EMEM 2% IBFS/Tris) before they were shipped to the laboratory. Total RNA was extracted from 200 *μ*L of the sample material using the automatic extraction instrument NucliSens easyMag (bioMérieux, Norge AS, Oslo, Norway) according to the manufacturer's instruction. Detection of influenza A virus was performed as described by the World Health Organization, The WHO Collaborating Centre for influenza at CDC Atlanta [20]. Specific detection of the HA gene of the pandemic (H1N1) 2009 subtype was carried out on influenza-A-positive samples as described by Robert Koch Institute [21]. Amplification was performed on a Stratagene Mx3500P (LaJolla, CA, USA) with Superscript III Platinum One-Step Quantitative RT-PCR system (Invitrogen, Paisley, UK). In addition, some samples were analyzed at DTU Veterinary (National Veterinary Institute, Technical University of Denmark) using an inhouse real-time RT-PCR for detection of pandemic (H1N1) 2009 virus (ref Statens Serum institut, Denmark, unpublished). One RT-PCR-positive sample was sufficient to confirm a herd as positive for pandemic (H1N1) 2009 virus.

 Full genome sequencing of isolate A/sw/Norway/02_11342/2009(H1N1) was carried out through a modified version of the WHO sequencing primers and protocol provided by the WHO Collaborating Centre for influenza in CDC, Atlanta, USA (28 April 2009) [22]. Briefly, 1 *μ*L extracted RNA was amplified using 46 primer pairs and a one-step RT-PCR Kit (QIAGEN, Oslo, Norway). The resulting overlapping amplicons, carrying primer-derived M13 terminal sequences, were sequenced with M13 primers using the ABI BigDye Terminator v1.1 Cycle Sequencing Kit and the ABI 3130 Genetic Analyzer (Applied Biosystems, Warrington, UK). Sequences were assembled using Sequencher v.4.9 (GeneCodes Corporation, Ann Arbor, MI, USA) and compared to related H1N1 virus sequences using BioEdit software [23].

### 2.4. Statistical Methods

Descriptive statistics and calculation of true population prevalence using confidence intervals were used. To measure the association between county prevalence and the pig density by county, we used simple linear regression or scatter plot analysis performed in Stata10.

## 3. Results

**Figure 1:**
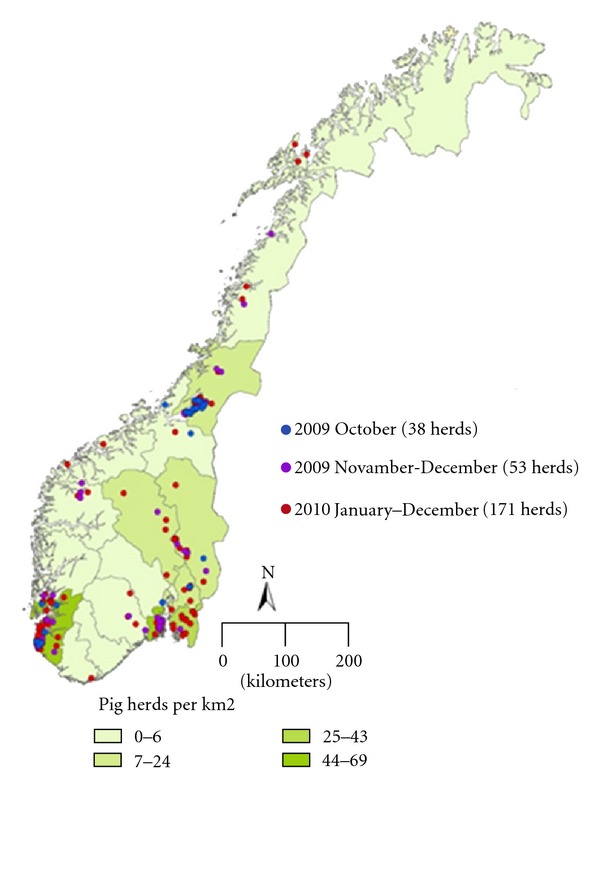
Map of Norway showing increasing number of herds tested positive for pandemic (H1N1) 2009 infection against the pig farming density by county (pig herds per km^2^).

After the introduction of pandemic (H1N1) 2009, the intensified surveillance (study 1) shows that 54 of the 114 herds tested were positive for pandemic (H1N1) 2009 virus by RT-PCR, and 55 of the 140 herds tested by serology were positive for antibodies against pandemic (H1N1) 2009. Altogether 91 herds tested positive from 10th of October to 31st of December 2009. In the 55 seropositive herds, 59% (range 5% to 100%) of the individual samples from each herd were positive. From the 54 RT-PCR-positive herds, an average of 65% (range 5% to 100%) of the individual samples from each herd were positive. Geographically, the infected herds were found throughout Norway (Figure 1). In 2010, two out of nine herds tested were positive for pandemic (H1N1) 2009 by RT-PCR. During the first six months of 2011, two out of five herds tested were positive by RT-PCR. These herds were sampled on suspicion of infection with SIV based on clinical signs in the pigs.

In study 2, a total of 2349 blood samples from 112 nucleus and multiplier herds were tested for antibodies against influenza A virus. Testing of the nucleus herds was completed by the 18th of December 2009, and the multiplier herds were finished on the 31st of January 2010. The surveillance shows that 27.1% of the nucleus herds and 34.4% of the multiplier herds were seropositive for pandemic (H1N1) 2009.

 Between six and 12 months after a herd was seropositive for antibodies against pandemic (H1N1) 2009 virus, blood samples were collected from slaughtered pigs born at least two months after the initial detection (study 3). In total, 1127 pigs from 38 herds were tested. Three of the 38 herds tested positive for antibodies against pandemic (H1N1) 2009 virus. The seropositive herds were multiplier herds located in the South West region where pig farming density is highest (Figure 1). Pigs from all the 13 previously positive nucleus herds selected for the study were found negative at this time. However, the intensified surveillance is still ongoing in the nucleus herds. By the end of July 2011, nine of the 13 nucleus herds were tested again. Pigs from five of these herds were positive for antibodies against pandemic (H1N1) 2009 virus, between 12 to 20 months of time after the primary infection in the herds.

 Within the 2009 routine surveillance of study 4, 4724 samples from 452 herds were tested for antibodies against SIV (Table 1). In total, 16 herds were found seropositive for pandemic (H1N1) 2009. Retrospectively, the earliest seropositive samples were collected from a herd in the county of Rogaland on the 30th of September 2009, suggesting that pandemic (H1N1) 2009 has been circulating in the Norwegian pig population since September 2009. The results from the surveillance program in 2010 show that 41% of 459 tested herds were seropositive (Table 1). The proportion of seropositive herds in different counties varied from 21% in the low pig density counties of Vestlandet to 57% in the high pig density county of Rogaland and Agder. Simple regression analysis shows that herd prevalence is positively correlated (*r* = 0.013, *p* = 0.023) with the pig density by county (Figure 1). Moreover, the surveillance program in 2010 shows that 42% (19 positive/45 tested herds) of nucleus and 44% (30 positive/68 tested herds) of multiplier herds were seropositive.

 Questionnaires with epidemiological information were available from 35 pig herds confirmed positive by laboratory testing (serology or PCR). In 60% (21/35) of these infected herds, there was evidence to suggest that humans were the most likely source of infections. In these herds, the farmer, farm worker, or the farmer's family members were diagnosed with pandemic (H1N1) 2009 (33% or 7/21) or had ILI just before or during the outbreak (66% or 14/21). Twenty percent (7/35) reported contact with another infected farm through humans or movement of infected animals as source of infection. One farmer of a positive herd had bought pigs from a positive herd and also had one farm worker with ILI during this period. Forty-nine percent (17/35) had reported that clinical signs were observed in the pigs. The most common clinical signs reported were coughing, fever, and loss of appetite, lasting for less than two weeks. No mortality was reported. Nineteen percent (8/43) of the herds from which epidemiological information was gathered tested negative although 3 of these herds had histories of contact with humans with ILI.

Full genome sequence analysis was carried out on nasal swab RNA from an infected pig in the index herd, as well as from the virus isolated from a sick staff member at the same farm. The sequences of these two full viral genomes were virtually identical (99.9985% identity) (Acc. no JQ253790-JQ253797). Moreover, the HA sequence showed at least 99.8% identity to other pandemic (H1N1) 2009 circulating in humans in Norway at the same time. The complete sequence further indicated that the virus had not acquired known resistance mutations in the neuraminidase gene.

## 4. Discussion

Pandemic (H1N1) 2009 virus was first detected in Norwegian pigs on the 10th of October 2009 [10]. As it was reported that the pandemic (H1N1) 2009 virus was transmitted rapidly between humans and also from humans to pigs, the Norwegian Food Safety Authority and the pig industry initiated biosecurity measures such as use of gloves and gauze masks to protect the Norwegian pig population from this virus. Following the detection of the index case, an intense screening based on detection of virus and antibodies against the virus was initiated. By the end of 2009, a total of 91 herds had tested positive for pandemic (H1N1) 2009 virus and/or antibodies against the virus. Our investigations showed that despite rapid implementation of control measures, the virus had in a short period of time been spread to pig herds throughout Norway, including herds that had high biosecurity and to closed herds.

 The first confirmed human case of pandemic (H1N1) 2009 virus in Norway was in May 2009 [24]. It was followed by a first minor wave of infected people during the Summer and a second major wave in the Autumn. Shortly after the onset of the Autumn wave, the first pig herd positive for pandemic (H1N1) 2009 virus was detected. Most Norwegian pig herds are small and family run. Hence, the farmer and family members must care for the pigs even when being ill. Many of the first positive pig herds had been in contact with people that were confirmed with pandemic (H1N1) 2009 virus infection or people with ILI [10]. Herd-to-herd transmission was ruled out because there was no history of contact between herds that were positive for the virus or were seropositive. It is also known that many people could be infected with pandemic (H1N1) 2009 without showing clinical symptoms. This suggests that infected humans, with or without clinical symptoms, were an important factor in the introduction and transmission of pandemic (H1N1) 2009 virus to the Norwegian pig population. Sequencing analysis of virus isolated from humans and from pigs indicated that almost identical viruses had infected both species [10]. Furthermore, the high prevalence in nucleus and multiplying herds, that normally have no or few contacts with other herds, indicates that humans, and not trade with pigs, were responsible for introduction of pandemic (H1N1) 2009 virus to Norwegian pigs.

 The national surveillance program for SIV in Norway shows that the first herd positive for antibodies against pandemic (H1N1) 2009 virus was sampled on the 30th of September 2009 [17]. As shown in Table 1, almost four percent of the herds that had been tested in this program in 2009 were seropositive. Of all the herds that were tested in 2010, 41% were seropositive. The results also show that in areas with high pig density, a high proportion of the herds were seropositive. This suggests virus transmission between pig herds, either by movement of pigs and people visiting infected herds. However, air transmission cannot be excluded [25]. The farm size and density of pigs in the area around the farm have been suggested to be important risk factors for influenza-seropositive farms [26]. Experimental studies have shown that pandemic (H1N1) 2009 virus is efficiently transmitted between pigs [11, 14, 15]. It is also known that other SIV subtypes are efficiently spread between pigs and herds [3].

 Norway has previously been free of SIV as documented by a national surveillance program for swine [17]. Although one multiplier herd was tested seropositive in 1998 for antibodies against influenza A H3N2 virus, follow-up studies for many years in this herd did not reveal new seropositive pigs, nor any spread of the virus in contact herds. Based on this, it was concluded that the pigs in this herd had probably been infected by a human-adapted H3N2 virus that did not have the capacity to be transmitted further between pigs [17]. Since the Norwegian pig population has been free of SIV, there are no cross-reactive antibodies to pandemic (H1N1) 2009 virus or any of the other SIV subtypes. This has probably contributed to the high prevalence of pandemic (H1N1) 2009 virus in the Norwegian pig population. It has been shown that European pigs naturally infected or vaccinated with European SIVs have cross-reactive hemagglutinating antibodies against pandemic (H1N1) 2009 virus [15, 16]. Moreover, natural infection with influenza virus stimulates mucosal immunity and cellular immune responses, responses that give partial protection against infection with an antigenetically unrelated strain [27, 28]. Surveillance of SIV in Europe prior to the emergence of pandemic (H1N1) 2009 virus showed that the three SIV subtypes ‘‘avian-like” H1N1, ‘‘human-like” H3N2, and ‘‘human-like” H1N2 were most often found in animals between 3- and 6 months old [29]. It should be noted that Norway is carrying out surveillance for SIV, including 4500–5000 samples from between 450 and 500 herds per year.

Consistent with other studies [8], there were mild or no clinical signs observed in herds that tested positive for pandemic (H1N1) 2009 virus. For example, in Australia, pigs naturally infected with pandemic (H1N1) 2009 virus only showed mild clinical signs. It was suggested that a lower infection pressure in natural infection as compared to experimental infection, as well as absence of other respiratory pathogens, could result in milder clinical signs [12]. In Norway, only a few pigs showed clinical signs like coughing, fever, and loss of appetite which are typical for SIV [3]. As Norway is free from most other viral respiratory diseases, presence of clinical signs like coughing would alert farmers who would notify their veterinarian or the authorities.

 Herds positive for pandemic (H1N1) 2009 virus in 2009 were tested again in 2010. The results show that there was no seroconversion in pigs born at least two months after the virus was first detected, indicating that there was no ongoing infection in the herds. A possible explanation may be that the Norwegian pig herds are small, and many herds practice batch farrowing of three, five and a half, or seven weeks intervals, with strict sectioning of the herds with all-in all-out system. However, results from the surveillance in 2011 show that some pigs born in 2010/2011 are seropositive, indicating an ongoing spread of the infection. It is not clear to what extent this is caused by new introduction from humans or continued virus circulation between susceptible pigs since 2009. Continuing surveillance is important to show whether the virus has become enzootic in the Norwegian pig population.

 There have been several reports of reassortant virus that have genes from pandemic (H1N1) 2009 virus and other SIV subtypes [30–33]. As long as Norway is free of other SIV subtypes, the risk of novel reassortant viruses spreading among pigs is low. However, reassortant viruses evolving in humans might be a risk for the Norwegian pig population. It is therefore important to continue the surveillance of SIV and genetic characterization of positive samples. Given the potential long-term presence of influenza virus in Norwegian pigs, it is also interesting to evaluate the economic impact of this disease and assess the cost effectiveness of new biosecurity measures or change in husbandry practices.

## 5. Conclusions

Pandemic (H1N1) 2009 virus was introduced to the Norwegian pig population in September/October 2009. The virus spread rapidly to a large proportion of all categories of herds all over the country, including closed, high-biosecurity nucleus herds. Humans seem to have been important for the introduction and spread of the infection. The clinical impact was mild, and in many infected herds, no clinical signs were observed. The ongoing national surveillance program will reveal whether the virus will become enzootic in the Norwegian pig population.

## Figures and Tables

**Table 1:** Surveillance programme for SI in Norwegian pigs in 2009 and 2010. Detection of pandemic (H1N1) 2009 specific antibodies by ELISA in different counties in Norway.

County	2009		2010	
	Number of herds tested	Number of seropositive herds (%)	Number of herds tested	Number of seropositive herds (%)
Troms and Finnmark	5	0 (0%)	5	2 (40%)
Nordland	20	0 (0%)	20	4 (20%)
Trøndelag and Møre	88	3 (3%)	88	48 (55%)
Vestlandet	11	0 (0%)	24	5 (21%)
Rogaland and Agder	117	11 (9%)	129	73 (57%)
Vestfold, Buskerud, Telemark	62	1 (2%)	59	20 (34%)
Østfold and Akershus	77	0 (0%)	69	20 (29%)
Hedmark and Oppland	72	1 (1%)	65	18 (28%)
Total Norway	452	16 (3.5%) (95% CI 2.2–5.7%)	459	190 (41%) (95% CI 37–46%)
